# Preservation of the relation between infarct characteristics and left ventricular remodeling following successful early revascularization for myocardial infarction: an observational study with contrast-enhanced cardiovascular MRI

**DOI:** 10.1186/1532-429X-13-S1-O69

**Published:** 2011-02-02

**Authors:** Marlon A Olimulder, Michel A Galjee, Jan van Es, Lodewijk J Wagenaar, Job van der Palen, Clemens von Birgelen

**Affiliations:** 1 MST Enschede, Enschede, Netherlands

## Introduction

Left ventricular (LV) remodeling following myocardial infarction (MI) is the result of complex interactions between various factors, including presence or absence of early revascularization. Cardiovascular Magnetic Resonance (CMR) imaging with contrast-enhancement (CE) permits assessment of myocardial tissue and LV dimensions and function, but the impact of early revascularization on the relationship between infarct tissue characteristics and LV remodeling has not yet been investigated.

## Purpose

We assessed the relation between infarct tissue characteristics and LV remodeling in a consecutive series of patients with vs. without successful early revascularization for prior MI who were examined with CE-CMR for clinical reason.

## Methods

CE-CMR data >1 month (median 11, range 1-425) after acute MI were obtained in 93 patients with vs. without successful early revascularization. Cine-CMR measurements included parameters of LV remodeling such as end-diastolic and end-systolic volumes, LV ejection fraction, and wall motion score index (WMSI). CE-CMR images were analyzed for infarct tissue characteristics including core, peri and total infarct size (IS), transmural extent, and regional scar scores.

## Results

**Figure 1:**
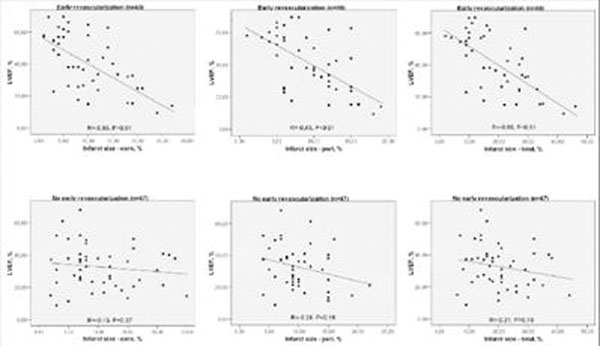
Univariate correlations between parameters of LV remodeling and infarct tissue characteristics in patients with (n=46) versus patients without (n=47) early revascularlization.

Effect modification by revascularization status was clear; therefore results were demonstrated separately. In early revascularized patients (n=46), infarct tissue characteristics correlated significantly with all parameters of LV remodeling (r=0.39-0.68; p<0.01). Multivariate analyses identified peri IS as the best predictor of all parameters indicating LV remodeling (R^2^=0.38-0.46). In patients without successful early revascularization (n=47), only a weak correlation between peri IS and WMSI was found (r=0.33; p=0.03). Figure 1.

## Conclusion

Only in successful early revascularized MI patients, a correlation between infarct tissue characteristics and LV remodeling was demonstrated with peri IS being the best determinant of remodeling. Our findings underline the importance of incorporating infarct tissue characteristics and the success of early revascularization when investigating LV remodeling.

